# Good Bronchial Hygiene Reaches the Left Lung: Successful Extubation in a Tetraplegic Patient With Spinal Cord Injury

**DOI:** 10.7759/cureus.28732

**Published:** 2022-09-03

**Authors:** Adriana Pascoal, Carolina Lourenço, César Pires, António Paiva, Ines M Vaz

**Affiliations:** 1 Physical Medicine and Rehabilitation, Centro de Medicina de Reabilitação da Região Centro - Rovisco Pais, Tocha, PRT; 2 Physical Medicine and Rehabilitation, Centro Hospitalar Vila Real, Vila Real, PRT

**Keywords:** mechanical insufflation-exsufflation, cough, bronchial hygiene, respiratory complications, spinal cord injury

## Abstract

Ineffective coughing affects bronchial hygiene and is a major contributor to respiratory complications after spinal cord injury (SCI). Mechanical insufflation-exsufflation (MIE) therapy increases inspiratory and expiratory flow to assist bronchial secretions clearance.

We present a case of a 67-year-old cervical SCI patient with lung infection and partial atelectasis in the lower left lung, associated with difficult ventilator weaning. About one day after the beginning of MIE therapy, an improvement of the atelectasis was verified. The patient was extubated six days after the beginning of bronchial hygiene with MIE therapy and safely transitioned to non-invasive ventilatory support.

## Introduction

Respiratory complications are a primary cause of morbidity and mortality after spinal cord injury (SCI) and can correlate with the denervation of the muscles involved in respiration and cough. Respiratory dysfunction severity is related to the neurological level of injury [1]. The cough reflex is maintained in cervical SCI. The ineffective cough is largely related to the weakness of the muscles of expiration. Spinal shock during the initial phase of SCI also impairs respiratory function due to mucous hypersecretion and flaccidity of the musculature below the level of the lesion [2].

The cough is a defense mechanism to protect lower airways, preventing respiratory tract infections, atelectasis, and other complications. Individuals with a peak cough flow below 270 L/min are at risk for secretion retention [3]. Ineffective coughing and secretion retention are major contributors to lower respiratory illness after SCI, especially during the acute phase [4].

Numerous techniques are used to enable clearance of bronchial secretions, like bronchoscopy, tracheal suctioning, manually assisted cough, air stacking, percussion, and postural drainage. Mechanical insufflation-exsufflation (MIE) therapy is a technique that increases inspiratory and expiratory flow to restore effective coughing and improve secretions mobilization [4]. MIE gradually applies positive air pressure (insufflation) to obtain a large volume of air within the lungs. The device then quickly reverses the flow of air by shifting to negative air pressure (exsufflation). The resulting high expiratory flow helps mobilize secretions out of the airway. To our knowledge, very few studies have examined the effectiveness of this technique in people with SCI.

In this case report, we discuss a cervical SCI patient with lung infection and partial atelectasis in the lower left lung, interfering with ventilator weaning. Our purpose is to show the magnitude of the potential benefits of MIE therapy in bronchial hygiene in these patients.

## Case presentation

A 67-year-old male patient slipped and fell from standing height in his home, followed by immediate loss of upper and lower limb sensory and motor function. Cervical magnetic resonance imaging (MRI) showed hyperintense intramedullary signal among C4 and C5 at T2-weighted imaging and central canal stenosis between C3 and C6. He underwent urgent C4-C6 laminectomy and C3-C6 fixation. He did not sustain another traumatic lesion, including traumatic brain injury.

Five days after the surgery, he was transferred to an intensive care unit (ICU) of another hospital, closer to his residency. The patient arrived feverish due to lobar nosocomial pneumonia with left inferior lung partial atelectasis.

He was empirically medicated with piperacillin/tazobactam since the surgery. Enteral feeding was delivered via a nasogastric tube. He required invasive ventilatory support after the surgery. He was ventilated with spontaneous ventilation mode, with pressure support (PS) of 12 cmH2O, positive end-expiratory pressure (PEEP) of 6 cmH2O, and fraction of inspired oxygen (FiO2) of 0.5.

At his time, the team debated if a tracheostomy was required, in view of a potentially difficult ventilation weaning. To help in the decision-making process, a physical medicine and rehabilitation (PMR) collaboration was requested the following day.

At PMR's first observation (day six of ICU care), the patient was calm and cooperative, showed no signs of higher function impairment, and denied being in pain. Neurological examination (following the International Standards for Neurological Classification of Spinal Cord Injury (ISNCSCI)) showed tetraplegia with the American Spinal Injury Association (ASIA) Impairment Scale (AIS) B, C4 neurological level of injury (sensory incomplete and motor complete), and the patient was still in the first phase of spinal shock. This motor (complete) level of injury implied a very high impairment risk in the patient’s ventilatory and coughing abilities and a susceptibility to lung atelectasis.

**Figure 1:**
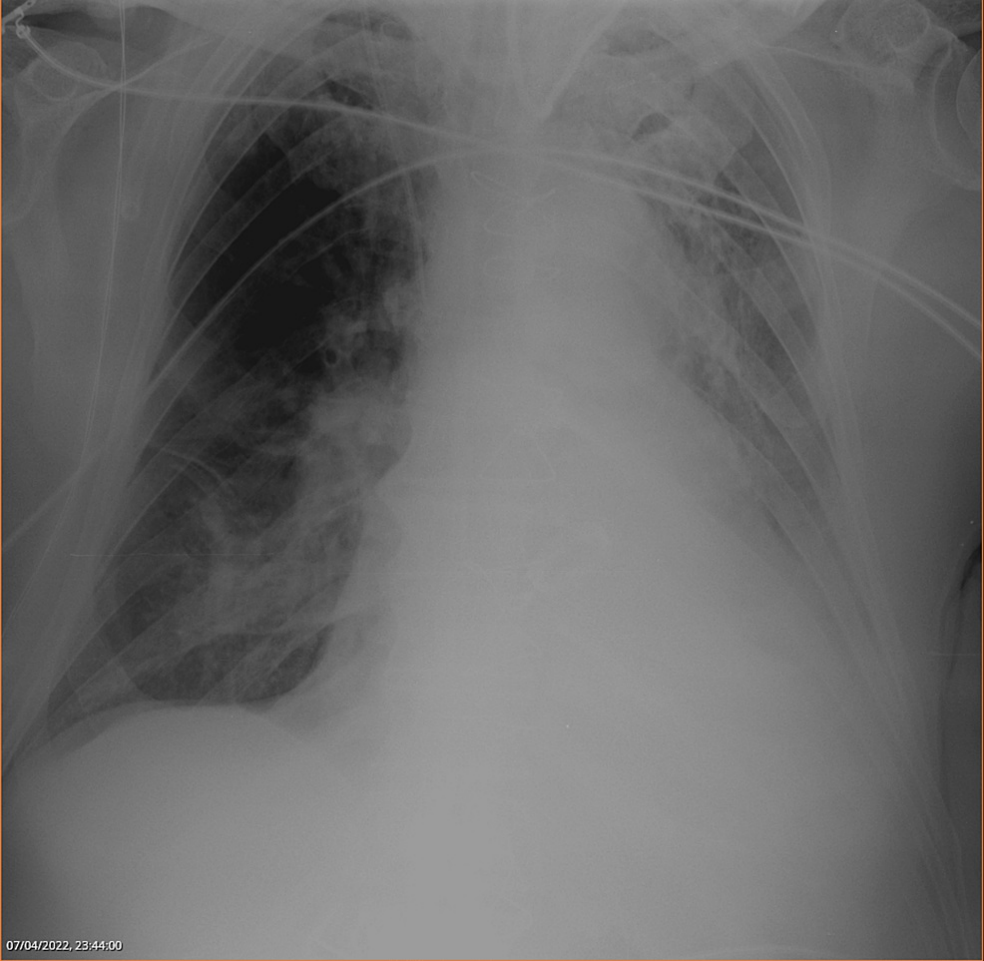
Left inferior lung partial atelectasis

Indeed, his pulmonary auscultation revealed a near absent vesicular murmur in the left inferior hemithorax. On top of that, the nursing team was having trouble performing adequate tracheal suctioning due to very adhesive and thick bronchial secretions. The chest X-ray of that day revealed no improvement of the left inferior lung partial atelectasis (Figure 1).

**Figure 2:**
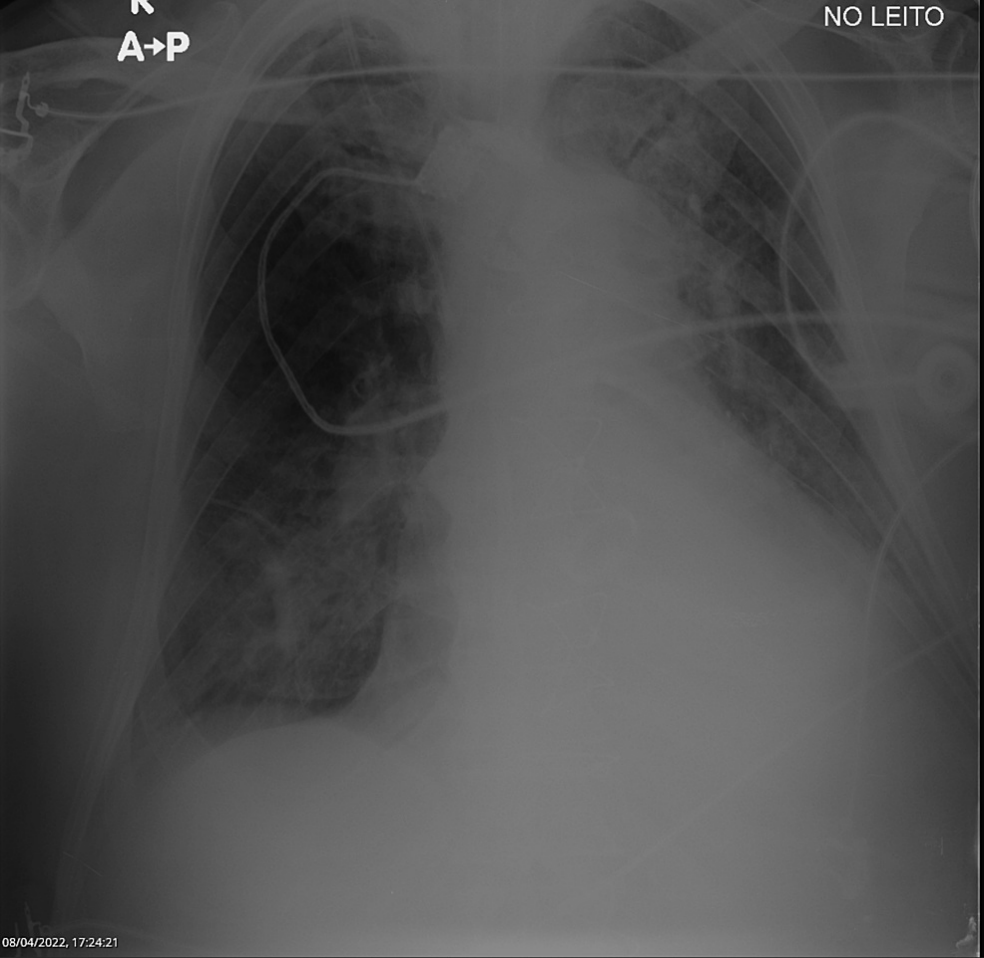
Partial reversion of the atelectasis after mechanical insufflation-exsufflation therapy

After confirming that there were no contraindications, we explained the procedure to the patient and obtained informed consent, and it was decided to trial the MIE with careful monitorization of arterial pressure, cardiac rhythm, and oxygen (O2) saturation. The following parameters were set: automatic mode with cough track, inspiratory pressure of 50 cmH2O for 2.4 seconds, and expiratory pressure of -60 cmH2O for three seconds. However, after five cycles of MIE, the secretions were still hard to expel, so an oscillation (frequency of 10 Hz and pressure variation of 5 cmH2O) was added during the inspiratory phase of the MIE, and five additional cycles were tried. After the second trial, the patient expelled a copious amount of mucopurulent secretions with a substantial pulmonary auscultation improvement in the left base. Afterward, he was prescribed three series of five cycles three times a day, with additional cycles if deemed necessary (i.e., O2 saturation < 95%). Another chest X-ray was repeated about one day after the sessions and it revealed a significant improvement, with a partial reversion of the atelectasis (Figure 2).

Bacteriological examination of sputum isolated *Escherichia coli* and *Serratia plymuthica*, both sensitive to piperacillin/tazobactam. After seven days of antibiotic therapy, there was a substantial clinical (sustained apyrexia) and analytic (C-reactive protein and leukocyte count reduction) improvement.

The patient was extubated six days after the beginning of bronchial hygiene with MIE. Right before extubation, he was ventilated with a spontaneous ventilation mode set, with PS of 10 cmH2O, PEEP of 6 cmH2O, and FiO2 of 0.31, and the arterial blood gas analysis demonstrated a pH of 7.46 (reference value: 7.35-7.45), serum bicarbonate (HCO3) of 24.9 meq/L (reference value: 22-26 meq/L), partial pressure of O2 of 188 mmHg (reference value: 75-100 mmHg), and partial pressure of carbon dioxide (CO2) of 35 mmHg (reference value: 35-45 mmHg). He safely transitioned to non-invasive ventilatory support (NIV), with no need for O2 support. The parameters during the day were volumetric NIV via a mouthpiece, touch trigger, total volume of 800 mL, inspiratory time of 1.2 seconds, and backup respiratory rate of 12 cycles per minute. The parameters for the night were bilevel positive airway pressure via a facial mask, inspiratory positive airway pressure (IPAP) of 8 cmH2O, expiratory positive airway pressure (EPAP) of 6 cmH2O, medium trigger, cycling into expiration at 25% of peak flow, minimal and maximal inspiratory time of 0.2 seconds and 1.5 seconds, respectively, and backup respiratory rate of 12 cycles per minute.

Three hours after extubation and non-invasive ventilatory support with volumetric mode, the arterial blood gas analysis showed a pH of 7.5 (reference value: 7.35-7.45), HCO3 of 28.9 meq/L (reference value: 22-26 meq/L), partial pressure of O2 of 63 mmHg (reference value: 75-100 mmHg), and partial pressure of CO2 of 37 mmHg (reference value: 35-45 mmHg). Four days after the extubation, he had a 90 L/min peak cough flow. So, inasmuch as he had an ineffective cough reflex, he had to maintain MIE therapy with three series of five cycles three times a day and when deemed necessary (O2 saturation < 95%).

## Discussion

A systematic review and meta-analysis that intended to describe extubation failure and associated risk factors in acute cervical SCI, concluded that the odds of unsuccessful extubation in a patient with complete cervical SCI were nearly three times that of a patient with an incomplete lesion [5]. Despite not being included in the formal statistical analysis, they state that it was possible that the potential ability of MIE to support the weaning of mechanical ventilation and post-extubation management had interfered with the variable results found [5].

The morbimortality of SCI patients with respiratory complications is frequently associated with ineffective cough flows [6]. There is some controversy regarding the ideal ventilation weaning protocol and tracheostomy indication in patients with compromised secretion mobilization. Intubation is realized to offer ventilatory support and assist secretion clearance. However, the endotracheal tube interferes with bronchial hygiene and the tracheostomy increases upper airway complications. Some authors advocate that in SCI patients that do not pass the ventilator weaning criteria, it is easier and better to extubate and transition them to non-invasive ventilation and MIE than to do an elective tracheostomy [6].

There is a high incidence of left-side pulmonary involvement in the acute SCI patient, which can be partially explained by the fact that suctioning misses the left main stem bronchus approximately 90% of the time [7].

It is certain that the respiratory system and consequently cough efficiency may be affected by SCI, but we still need to know the most effective treatment strategy. However, the comparison between airway clearance techniques in SCI patients is a poorly researched area. Randomized controlled trials (RCTs) of quality are difficult to carry out in the airway management area because of the ethical risk to deny the best possible treatment to a control group [8]. Nevertheless, one RCT evaluated cough strength through measurements such as peak cough flow and found that MIE is more effective at restoring cough than using only manual techniques during the acute phase post SCI [9].

Not less important, a study demonstrated that SCI patients report a more positive experience when using MIE versus those that experience endotracheal suctioning. These patients reported that MIE was significantly less irritating, painful, tiring, and uncomfortable [10].

## Conclusions

Particular attention should be given to the respiratory system function in patients with SCI, including secretions management. Greater effort should be made to optimize the respiratory management in the acute phase of these patients, with more investigation and education regarding effective and non-invasive therapies like MIE.

All in all, we think that MIE can be a very important tool in preparing patients for extubation, since it provides a way of dealing with bronchial secretions and preventing atelectasis, especially in the left lobe.
